# Application of Wavelet Packet Entropy Flow Manifold Learning in Bearing Factory Inspection Using the Ultrasonic Technique

**DOI:** 10.3390/s150100341

**Published:** 2014-12-26

**Authors:** Xiaoguang Chen, Dan Liu, Guanghua Xu, Kuosheng Jiang, Lin Liang

**Affiliations:** 1 School of Mechanical Engineering, Xi'an Jiaotong University, Xi'an 710049, China; E-Mails: chenxiaoguang2008@163.com (X.C.); ghxu@mail.xjtu.edu.cn (G.X.); jiangkuosheng333@stu.xjtu.edu.cn (K.J.); lianglin@mail.xjtu.edu.cn (L.L.); 2 State key Laboratory for Manufacturing Systems Engineering, Xi'an Jiaotong University, Xi'an 710049, China

**Keywords:** rolling bearing, piezoelectric ultrasonic transducer, wavelet packet entropy flow manifold learning, bearing factory quality evaluation

## Abstract

For decades, bearing factory quality evaluation has been a key problem and the methods used are always static tests. This paper investigates the use of piezoelectric ultrasonic transducers (PUT) as dynamic diagnostic tools and a relevant signal classification technique, wavelet packet entropy (WPEntropy) flow manifold learning, for the evaluation of bearing factory quality. The data were analyzed using wavelet packet entropy (WPEntropy) flow manifold learning. The results showed that the ultrasonic technique with WPEntropy flow manifold learning was able to detect different types of defects on the bearing components. The test method and the proposed technique are described and the different signals are analyzed and discussed.

## 1. Introduction

Bearings play an important role and have been applied in a variety of fields in modern industry. The stability and reliability of bearings depends crucially on materials, lubrication, environment and maintenance techniques. Evaluation of the bearing factory quality is necessary before delivery to ensure safe machinery operation and avoid unexpected breakdowns. In recent years, various sensors and different advanced signal processing techniques have been tried and developed for this purpose. For example, vibration analysis tools, such as accelerometers and eddy current sensors, represent the most common methods to diagnose bearings [1]. There are varieties of advanced signal processing techniques such as wavelet transforms [2], Principal Component Analysis [3], manifold learning [4], minimum entropy deconvolution [5], spectral kurtosis [6], envelope analysis [7], and so on. The replacement of accelerometers has been considered and other sensors studied, such as acoustic emission (AE) sensors [8–10]. Acoustic emission signals have demonstrated superior to vibration signals in the early stages of bearing failure. The acoustic emission sensor can easily identify the defects on the outer race, but it fails to distinguish defects on the inner race. Infrared thermography [11] has been also investigated in recent years, but the sensors are hard to install and the tests are hard to carry out. Recently, Dadouche [12–14] showed that an air-coupled ultrasound transducer is very effective in extracting fault features from different bearing components. These results were very promising. The abovementioned studies are representative techniques to show the rising interest in using novel sensors and algorithms to perform better diagnosis of bearing faults in order to develop effective prognostic tools.

The ultrasonic technique has recently been proposed as an effective tool for condition monitoring of ball bearings. Unlike the traditional sensors, there exists literature work demonstrating the feasibility and capability of the piezoelectric ultrasonic transducer (PUT) to detect bearing defects [14–16]. However, there is a lack of literature on the application of PUT for bearing diagnosis using advanced signal processing techniques. For instance, the authors of [14] tried to use PUT and showed PUT was a unique sensor for its capacity of detecting outer race defects on the bearing using a simple PSD analysis of the raw signal, which indicated the feasibility of PUT to detect bearing faults. However, it was pointed out in [14] that analysis of the PUT sensor signal could not reveal any distinguishable data with time and wavelet analysis. In this study, the authors investigate the use of piezoelectric ultrasonic transducers and develop relevant advanced signal processing techniques for the evaluation of bearing factory quality. Our purpose in this study is to develop the PUT and suitable processing techniques for the evaluation of bearing factory quality before delivery. Wavelet packet entropy flow manifold learning was developed as the signal processing technique, which showed its effectiveness in isolating defect signatures.

## 2. The Ultrasonic Technique for Bearings

Ultrasound is defined as sound waves that have frequency above 20 kHz. Many machine components, especially rotating components, emit consistent ultrasound patterns. These ultrasonic features can be received and identified by ultrasonic transducers, and changes in these features when components begin to wear or deteriorate can be distinguished [15].

It is worth noting that the AE technique also deals with signals in the high frequency range for bearing condition monitoring. However, the AE technique focuses on the frequency range of 100 kHz to 1 MHz, while the ultrasonic technique covers 20 kHz to 100 kHz [15,16]. Signal processing methods such as events, ring down counts and peak amplitude are usually employed in the AE technique, while in the ultrasonic technique, listening to the sound features and the heterodyne technique are usually used.

## 3. Test Rig Description

**Figure 1.**
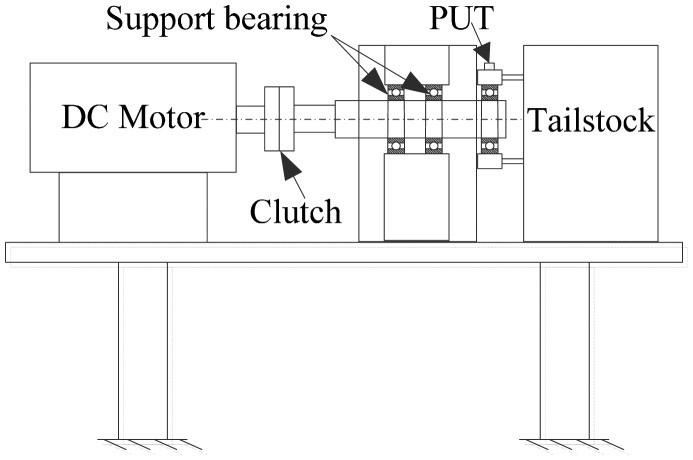
Schematic diagram of the test rig made up for 6308 ball bearings.

The schematic diagram of the test rig made up for 6308 bearings is shown in Figure 1. It consists of a shaft supported on three greased ball bearings, two support bearings and one test bearing. The shaft speed was kept constant during tests with the inner ring free to rotate under light load. The PUT used for this study was P5Ф8 (SIUI, Inc., Shantou, China) with a frequency response range up to 2.5 MHz. Although the PUT is a contact sensor that usually used in the pulse-echo mode for structural analysis, only the receiver mode was used in this study.

**Figure 2.**
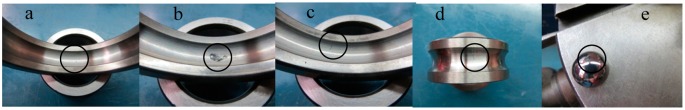
Implanted bearing defects: (**a**) bruise on the outer race; (**b**) corrosion on the outer race; (**c**) scratch on the outer race; (**d**) scratch on the inner race; (**e**) scratch on the ball.

The defects on different bearing components, the ball, inner, and outer races separately, were intentionally created before they were assembled, simulating damages that would evolve from fatigue cracks or other relevant defects (Figure 2). Notably, the defects were very slight.

**Figure 3.**
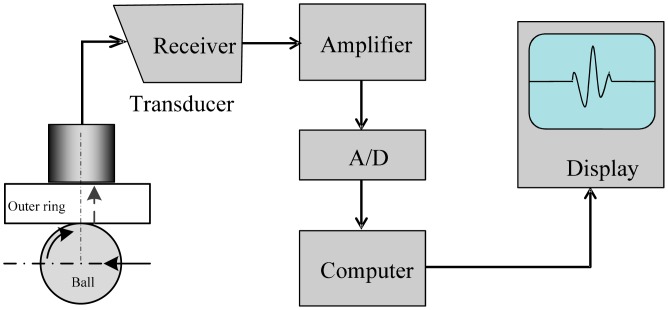
Block diagram of the PUT measurement system.

As shown in Figure 2, a piezoelectric ultrasonic transducer was used to record the signals of the healthy as well as the defective bearings. Figure 3 shows a block diagram of the PUT measurement system.

## 4. Signal Processing

### 4.1. Time Domains

The root mean square (RMS) value, as the most usual time domain analysis method, is given by:

(1) $$RMS=\sqrt{\frac{1}{N}\sum_{n=0}^{N-1}{\left(x(n)-\bar{x}\right)}^{2}}$$

where *x̅* is the mean value of the signal.

### 4.2. WPEntropy Characteristics

The wavelet packet (WP) coefficients of signal *x*(*t*) can be calculated as below [17]:

(2) $$p_{j}^{n}(\text{k})=\langle x,W_{j,k}^{n}\rangle =\int_{-\infty }^{\infty }x(\text{t})W_{j,k}^{n}{(\text{t})\text{d}}_{\text{t}}$$

where $p_{j}^{n}(\text{k})$ denotes the *n*-th set of WP coefficients at the *j*-th scale parameter and *k* is the translation parameter, and *W* is the WP function.

Traditional WPT-based (wavelet packet transform) measures [18] include entropy, energy, standard variation, *etc*. WPEntropy characteristics focuses on the entropy characteristics distributed on the WP nodes.

Mathematically, for a discrete signal *x*(*t*) with *N* data points, assuming *N* = 2*^n^*^0^, the wavelet coefficients of node (*j*, *n*) can be denoted by $\left\{P_{j}^{n}\left(k\right),k=1,2,\ldots ,2^{n0-j}\right\}$. The signal entropy contained in the WP node (*j*, *n*) is calculated as:

(3) $${\text{Entro}}_{\text{j}}^{n}=-\sum_{k=1}^{2^{n0-j}}{\left[p_{j}^{n}(\text{k})/\sum_{k=1}^{2^{n0-j}}p_{j}^{n}(\text{k})\right]}^{2}\log{\left[p_{j}^{n}(\text{k})/\sum_{k=1}^{2^{n0-j}}p_{j}^{n}(\text{k})\right]}^{2}$$

Following the entropy, define the average entropy at each WP node as:

(4) $${\text{AEntro}}_{\text{j}}^{n}=\frac{1}{2^{\text{n}0-j}}{\text{Entro}}_{\text{j}}^{n}$$

Then the average entropy vector for the *j*-th level can be written as below:

(5) $${\text{AEntro}}_{\text{j}}={\left[\text{AEntr}o_{j}^{1}\right]}_{1\times 2^{n0-j}}\cdots {\left[{\text{AEntro}}_{j}^{n}\right]}_{1\times 2^{n0-j}}\cdots {\left[{\text{AEntro}}_{j}^{n}\right]}_{1\times 2^{n0-j}}\in R^{1\times 2^{n0}}$$

For different levels, put the average power vectors at levels 0 to *J* together, then deliver the average entropy matrix as follows:

(6) $$\text{AEntro}={\left[\begin{array}{cccc} {\text{AEntro}}_{0}^{T} & {\text{AEntro}}_{1}^{T} & \cdots & {\text{AEntro}}_{\text{J}}^{T} \end{array}\right]}^{T}\in R^{\left(J+1\right)\times 2^{\text{n}0}}$$

Reorganize the average entropy vector by reducing the dimension of the matrix *AEntro*:

(7) $${\text{RAEntro}}_{\text{j}}={\left[{\text{AEntro}}_{j}^{1}\right]}_{1\times 2^{J-j}}\cdots {\left[{\text{AEntro}}_{j}^{n}\right]}_{1\times 2^{J-j}}\cdots {\left[{\text{AEntro}}_{j}^{n}\right]}_{1\times 2^{J-j}}\in R^{1\times 2^{J}}$$

Then finally we get the WPEntropy matrix:

(8) $$\text{WPEntro}={\left[\begin{array}{cccc} {\text{RAEntro}}_{0}^{T} & {\text{RAEntro}}_{1}^{T} & \cdots & {\text{RAEntro}}_{\text{J}}^{T} \end{array}\right]}^{T}\in R^{\left(J+1\right)\times 2^{J}}$$

where *RAEntro_J_* is called the WPEntropy vector that corresponds to the WP nodes entropy at the final level *J* of the WPT.

### 4.3. LTSA (Local Tangent Space Alignment)

The above entropy flow characteristics among WP nodes can be revealed by manifold learning. The WPEntropy flow characteristics can be seen as nonlinear structure contained in the WPEntropy matrix. There are many manifold learning techniques, such as Isometric Mapping (ISOMAP), Local Linear Embedding (LLE), and LTSA. Compared to ISOMAP and LLE, LTSA is superior in the signals with non-uniform distributed noise and in obtaining intrinsic data structure [19].

LTSA supposes local geometric characteristics could be expressed by sample tangent space in the neighborhood, and therefore the coordinates of global manifold are constructed by arranging all neighbor local tangent space matrixes. The LTSA algorithm can be summarized as follows:

(1) Local neighborhood selection. Let *X_i_*= [*x_i_*_1_,…, *x_ik_*] be the local neighborhood matrix of *x_i_*, where *x_ik_*is the *k*-th neighborhood point nearest to *x_i_*.
(2) Local neighborhood linear mapping. For samples of every local neighborhood matrix, construct a normalized centre matrix $X_{i}-\bar{x_{\iota }}e^{T}$, and calculate the eigenvectors of the *d* largest eigenvalues to construct a new eigenmatrix *Q_i_*. The local coordinate system is defined by:
  (9) $$\Theta_{i}=Q_{i}^{T}X_{i}\left(I-ee^{T}/k\right)=[\theta_{1}^{\left(i\right)},\ldots ,\theta_{k}^{\left(i\right)}]$$
  where $\theta_{j}^{\left(i\right)}=Q_{i}^{T}\left(x_{ij}-\bar{x_{\iota }}\right)$.
(3) Local coordinate system arrangement. Define local weight matrix *W* = *diag*(*W*_1_,…,*Wn*), where $W_{i}=\left(I-ee^{T}/k\right)\left(I-\Theta_{i}^{+}\Theta_{i}\right)$. Then global permutation matrix *B* = *SWW^T^S^T^*, where *S* = [*S*_1_,…, *S_N_*] and *S_i_* is the selection matrix that *S_i_* = [*x_i_*_1_,…, *x_ik_*].
(4) Calculate eigenvectors of permutation matrix. Calculate the eigenvectors *u*_2_,…, *u_d_*_+1_,which correspond to the 2nd to *d* + 1th smallest eigenvalues. Then *T* = [*u*_2_, *u*_3_, …, *u_d_*_+1_]*^T^* = [*t*_1_, *t*_2_, …, *t_N_*] is the embedded result.

### 4.4. WPEntropy Manifold Feature

**Figure 4.**
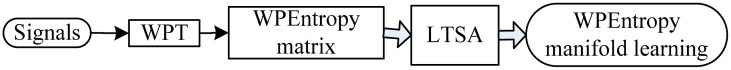
Scheme of the WPEntropy manifold feature extraction.

LTSA can catch the nonlinear flow signature of the major entropy. Different kinds of signals will have different nonlinear WPEntropy flow patterns, so they will contribute different features to characterize the vibration pattern. Given a set of training signals, the basic idea of the WPEntropy manifold feature extraction is illustrated in Figure 4.

## 5. Data Analysis

**Figure 5.**
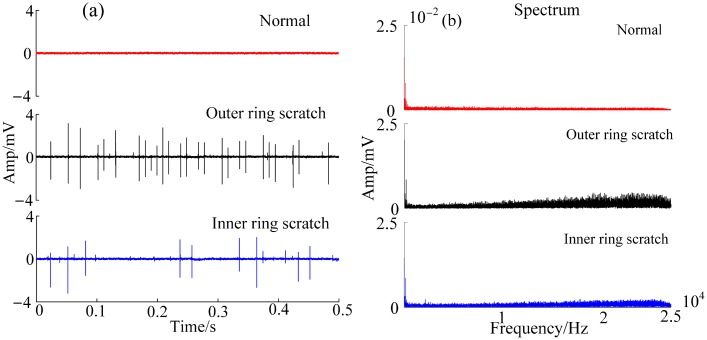
PUT data, normal, scratch on the outer race and the inner race: (**a**) raw signal; (**b**) spectrum.

This section discusses the experimental data obtained from the test bearings with various implanted defects. The emphasis was on assessing the fault detection ability of the PUT. For the same defective bearings, Figure 5 illustrates the raw signal and its spectrum acquired with the PUT. It can be seen that there are spikes in the raw signals of the defective bearings, but there are almost no spikes in the raw signal of the normal bearing. After calculating their RMS, the RMS with the outer-race-scratch bearing is 1.2612 × 10^−4^ V, the RMS with the inner-race-scratch bearing is 0.9147 × 10^−4^ V, and the RMS with the normal bearing is 3.1073 × 10^−5^ V. Obviously, the RMS with the normal bearing is not of the magnitude as the RMS with the defective bearings, which paves the way to the fast detection of bearing health monitoring. However, in the spectrum as shown in Figure 5b, we cannot get more information about the defects. Therefore, a suitable signal process method should be developed to make the PUT signal effective.

In general, it is difficult for the accelerometer to extract the defect signature by spectrum for the defects on the inner race due to the poor signal-to-noise ratio and attenuation [12,15]. Normal and abnormal bearings are easily distinguished with the PUT, but it is hard to identify which kind of defect it is and where it occurred. Therefore, we use the WPEntropy manifold feature in the next step to identify what type of defect is involved and where it occurred with the PUT signal.

We first chose normal bearings and three kinds of defective bearings with scratches on different components, the outer, inner race, and ball, respectively. The PUT was used to record the signals of the normal as well as the defective bearings. The obtained dataset contains 40 training samples for each bearing condition. Ten time domain parameters of each sample were employed for the WPEntropy flow manifold learning covering mean value, peak value, RMS, root, variance, kurtosis, peak factor, margin factor, skewness, and impulsion index.

**Figure 6.**
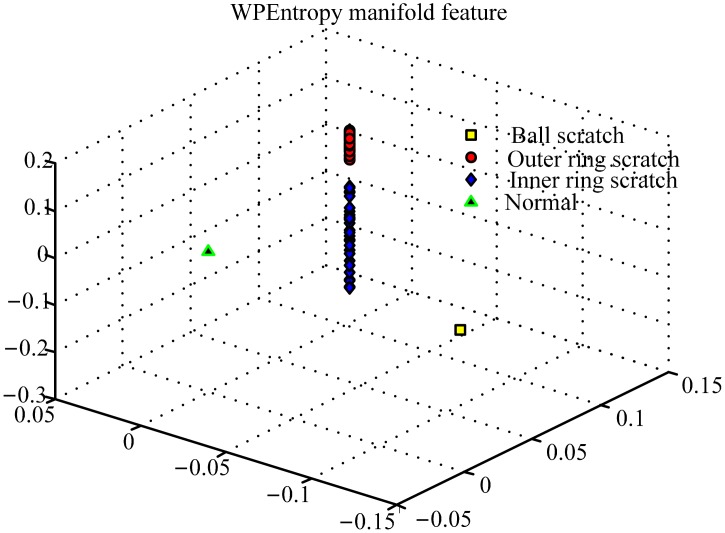
Representation of training samples of bearing data by WPEntropy manifold learning for scratch on different components.

With the algorithm of WPEntropy flow manifold learning described in Section 4, the results are shown in Figure 6. The WPEntropy manifold representation in Figure 6 shows the samples in each class almost gather at one point, which intuitively exhibits the excellent merit of the WPEntropy manifold feature in clustering. Therefore, the present method is valuable for PUT signal classification.

**Figure 7.**
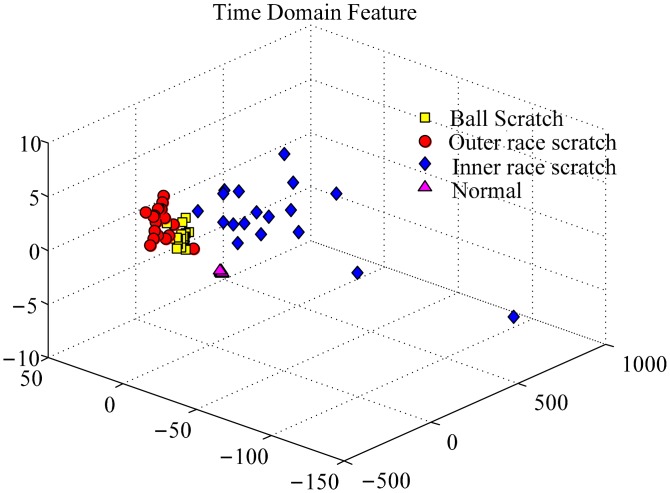
Representation of training samples of bearing data by PCA for scratch on different components.

To investigate the effectiveness of the WPEntropy manifold learning for PUT signal classification, Principal Component Analysis (PCA), which is a linear algorithm, is used for analysis of the same dataset. As shown in Figure 7, it can be found that it is hard to distinguish the three defective bearings in comparison with the WPEntropy manifold learning. The result confirms the effectiveness for the WPEntropy manifold feature for PUT signal classification.

**Figure 8.**
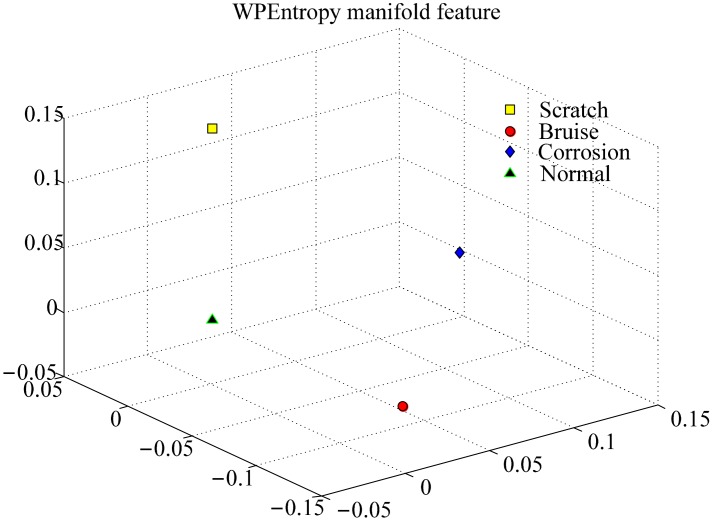
Representation of training samples of bearing data by WPEntropy manifold learning for different defects on the outer race.

**Figure 9.**
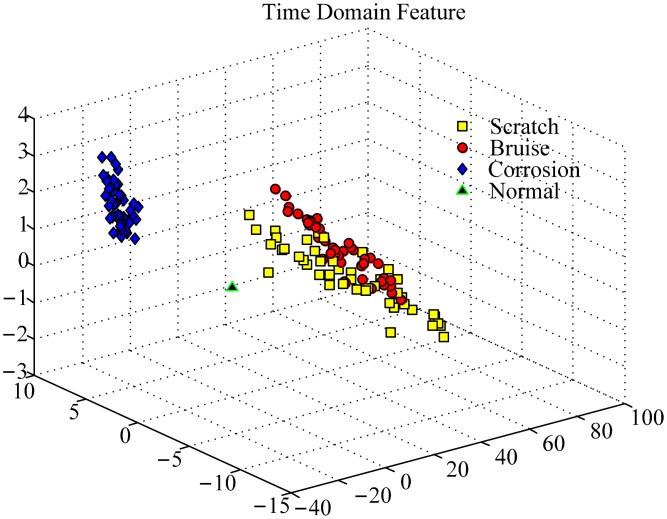
Representation of training samples of bearing data by PCA for different defects on the outer race.

To further verify the effectiveness of the WPEntropy manifold learning for PUT bearing defect classification, three different defects on the outer race were tested, including scratch, corrosion, and bruise, as shown in Figure 3. Usually, it is very difficult to distinguish these defects on the same component for accelerometers. With the same signal processing, Figure 8 shows the samples in each class almost gather at one point too, but the samples are also hardly distinguished with PCA as shown in Figure 9. The results also confirm the excellent performance for the WPEntropy manifold learning for PUT signal classification.

## 6. Discussion

The main contribution of this paper is to explore the use of PUT for bearing health monitoring and relevant suitable signal processing techniques. There are traditional sensors for bearing fault detection, such as the accelerometer. Unlike the traditional sensors, the PUT data can easily distinguish between healthy bearings and defective bearings, so it is beneficial for fast bearing health monitoring. The above studies have shown the valuable effectiveness of the PUT.

In this paper, only the time domain indexes with the selected WPEntropy manifold learning technique were employed for classification of the PUT data. The spectrum of the PUT raw signal showed no available information for identifying the defects, therefore, advanced frequency domain analysis methods or other signal processing techniques, such as spectral entropy and wavelet transforms should be considered for a better bearing defect identification.

## 7. Conclusions and Future Work

In this paper, the authors studied the ability of the PUT to detect and identify faulty bearings with different defect forms and locations. The results with the PUT were promising. Moreover, the WPEntropy manifold learning was developed as the PUT signal processing technique. The main conclusions are as follows:

1. The PUT has the potential and ability to identify bearing defects, especially on the inner race and different kinds of defects on the ball.
2. WPEntropy manifold learning demonstrates its effectiveness in isolating the nonlinear bearing defect signatures using the PUT with rig data.
3. By the WPEntropy manifold learning method, defects on different bearing components as well as different defects on the same component can be easily distinguished for PUT signal classification.

The next step will be to study how to extract the characterized frequency of bearing defects with the PUT data and the effect of load and speed on the ability of the PUT to detect different faults. Additional hardware will be used to filter out the noise and record the ultrasound signal. Moreover, the pulse-echo mode (PUT) will be investigated in detail for rolling bearing health monitoring. The pulse-echo mode and receiver mode of PUT will be coupled with fault models towards developing a new diagnostic tool for bearings in service.
